# The effect of 12-week combined balance and plyometric training on dynamic balance and lower extremity injury risk in college dancers

**DOI:** 10.3389/fphys.2025.1501828

**Published:** 2025-02-06

**Authors:** Yuqi Yan, Park Seoyoung, Heo Seomyeong, Yi Zhao

**Affiliations:** ^1^ Dance College, Sichuan Normal University, Chengdu, China; ^2^ School of College of Art and Physical Education, Hanyang University, Seoul, Republic of Korea

**Keywords:** dynamic balance, plyometric training, balance training, lower extremity injury risk, college dancers dynamic balance, college dancers

## Abstract

**Background:**

Dancers face significant physical demands and are at high risk for lower extremity injuries due to the complexity and intensity of their movements, which require strong dynamic balance. Improving dynamic balance through training can potentially enhance performance and reduce injury risk.

**Objective:**

This study aimed to investigate the effects of a 12-week combined balance and plyometric training program (BP) compared to plyometric training alone (PL) on dynamic balance and lower extremity injury risk among college dancers.

**Methods:**

A total of 30 female college dancers were randomly assigned to either the BP group (n = 15) or the PL group (n = 15). Both groups participated in a 12-week training program, with the BP group engaging in both balance and plyometric exercises, and the PL group performing only plyometric exercises. Dynamic balance was assessed using the Dynamic Posture Stability Index (DPSI). Lower extremity injury risk was evaluated using the Limb Symmetry Index (LSI) and Center of Pressure (COP) measurements, pre- and post-intervention.

**Results:**

The BP group showed significant improvements in dynamic balance compared to the PL group, with a reduction in DPSI values (DF-DPSI: p < 0.001, partial η^2^ = 0.625; DL-DPSI: p < 0.001, partial η^2^ = 0.559). Additionally, the BP group showed significant reductions in COP displacements, particularly in the anterior-posterior direction (DF-COPAP: p < 0.015, partial η^2^ = 0.101; DL-COPAP: p = 0.019, partial η^2^ = 0.094). The BP group also demonstrated greater improvements in LSI-3C and LSI-6, which reflect dynamic stability (LSI-3C: p < 0.001, partial η^2^ = 0.229; LSI-6: p = 0.006, partial η^2^ = 0.128).

**Conclusion:**

The 12-week combined balance and plyometric training program was more effective than plyometric training alone in improving dynamic balance and reducing lower extremity injury risk in college dancers. This combined training approach is recommended for improving performance and preventing injuries in dancers.

## Introduction

Professional dancers are not only artists but also athletes. Dance performance is not a single art activity, but it is a complex phenomenon depending on multiple elements with both direct and indirect effects during dance performance, such as physically fitness. The intense physical demands of dancing expose dancer’s feet to a high risk of injuries such as hallux valgus, metatarsal injury, and subsequent ankle pain (Furia et al., 2010). Additionally, “dance injury” is common among dancers because of the high-intensity training required and the technical discipline and rigour needed for dance performance (Shah et al., 2012). Unlike athletes in other sports, dancers engage in more fluid and complex movements that require superior dynamic balance, with an injury incidence of up to 95% over a dancer’s lifetime (Byhring and Bø, 2002). Dancing involves many quick and slow motions, including acceleration, deceleration, rotation, and single leg support (Clarke et al., 2021), making dynamic balance a key component of dance performance (Koutedakis and Jamurtas, 2004). This unique demand for balance in dance—different from that in sports such as football or basketball—requires a more specific training approach. Moreover, dynamic balance ability is linked to lower-limb injuries. Therefore, strategies aiming to improve dynamic balance hold great promise to improve dance performance and reduce injury risk among dancers.

Plyometric training (PT) is one such strategy, which consists of motions related to the eccentric-concentric contraction cycle of muscles, also known as the stretch-shortening cycle (SSC) (Markovic and Mikulic, 2010) (e.g., depth jump and continuous jump (Stojanović et al., 2017)). PT is widely used in the training of athletes, as it can improve strength and power performance. Several studies have examined the effects of PT on strength. Recent studies have shown that compared to traditional resistance training (RT), PT can lead to comparable or even better enhancement of the performance of athletes (Ziv and Lidor, 2010a; Asadi et al., 2016) by improving their power and strength. The PT has also shown positive effects in enhancing dynamic balance and proprioception in athletes. For instance, Alikhani et al. (2019) observed that a 6-week PT could significantly improve dynamic balance and knee proprioception in female badminton players, which can ultimately prevent injury among participants. These findings suggest that PT could also be beneficial for dancers, whose lower limbs are constantly subjected to dynamic loads and high-impact movements.

However, while PT has been widely studied, the combined effects of PT and balance training specifically in dancers remain unclear. Recently, a combined intervention has been developed by concurrently implementing two types of training programs. The combined training can simultaneously improve multiple underlying domains contributing to dynamic balance, thereby inducing greater effects on performance compared to the intervention using only one training type. Studies demonstrated that combined training can significantly improve the strength, balance, and change of direction (COD) ability of basketball, football, and badminton players (Makhlouf et al., 2018; Muehlbauer et al., 2019) compared to the intervention using only one type of training. For instance, Guo et al. implemented the combined training of PT and balance (PB) in badminton players. This PB training consisted of depth and continuous jumps with balance exercises on unstable platforms. The results of this study showed that PB can significantly improve COD performance compared to PT only (Guo et al., 2021a). However, this combined approach has not been tested in dancers, who face a unique set of biomechanical challenges.

Therefore, the present study aimed to examine the effects of a 12-week combined training on dynamic balance and lower extremity injury risk among college dancers. This study is novel because it focuses on dancers’ specific movement patterns and biomechanical needs, exploring how combined PT and balance training could uniquely enhance their dynamic balance. We hypothesized that a combined training protocol would induce a greater increase in parameters pertaining to dynamic balance and decrease the injury risk compared to PT.

## Materials and methods

### Subjects

**FIGURE 1:**
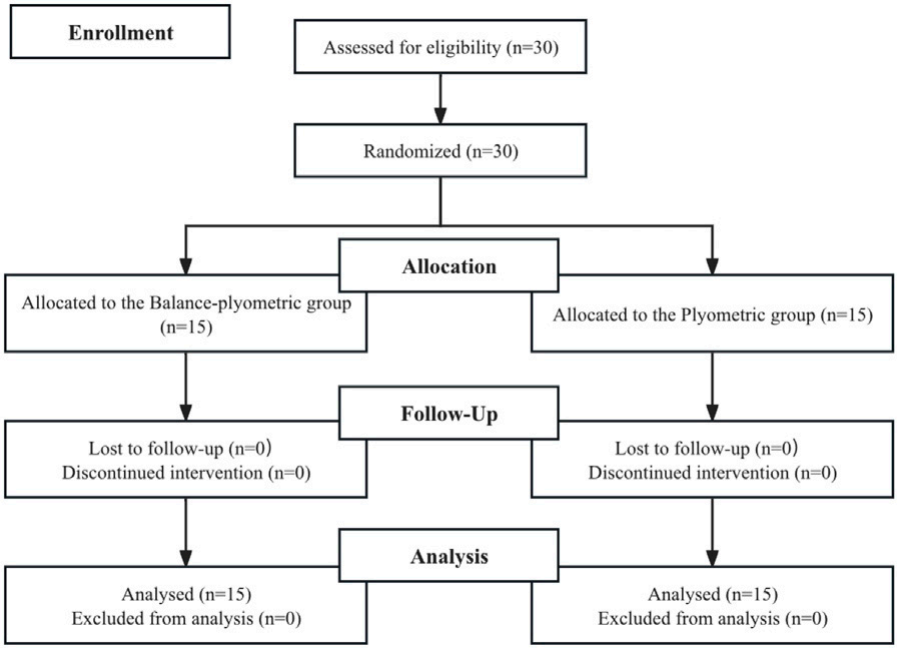
Flow chart of the progress through the phases of the study according to the CONSORT statements.

The sample size of this study was 30 participants, determined using GPower (version 3.1.9.7; Franz Faul, University of Kiel, Kiel, Germany). These calculations were based on an α error probability of 0.05, a power (1-β error probability) of 0.8, an effect size (ES) of 0.4, and a test family encompassing F-tests and analysis of variance (ANOVA), specifically focusing on repeated measures and within-between interaction (Beck, 2013). A total of 30 female college dancers volunteered to participate in this study considering 15%–20% attrition rate during the test and intervention. Participants were randomly assigned into a BP group (n = 15, age: 22.51 ± 3.92 years, height: 167.41 ± 6.12 cm, weight: 52.87 ± 6.45 kg, and training experience: 5.4 ± 1.4 years) and a PL group (n = 15, age: 22.90 ± 3.85 years, height: 168.82 ± 4.69 cm, weight: 51.29 ± 5.91 kg, and training experience: 4.4 ± 1.3 years) using a RAND function (1 and 2) (Microsoft Excel 2019) (Figure 1).

Study participants were recruited from April 1 to 30 April 2024, and the experiment was conducted from May 1 to August 1. The inclusion criteria were college dancers (1) who were female, (2) were versed in resistance and plyometric training skills, and (3) had the capability and intention to complete the 12-week training program including the exercise and testing. The exclusion criteria were participants (1) who had sustained serious injuries to their lower limbs, such as the anterior cruciate ligament, hamstring, meniscus, and ankle, or had developed any medical or orthopedic problems in the last 3 years and (2) were unable to perform plyometric training. Participants provided written informed consent after being informed about the potential benefits and risks associated with data collection. They were instructed to maintain their regular diet, avoid additional nutritional supplements, and consume caffeine-free beverages during the study. The study received approval from the Shandong Normal University Institutional Research Commission (Approval number: 2023105) and adhered to the guidelines of the Declaration of Helsinki.

### Procedures

All experimental training programs were conducted along with a weekly technical training routine. Participants from the BP and PL groups followed a balance training combined with a plyometric training program (40 min of plyometrics and 20 min of balance training) three times per week with 24–48 h of recovery between each training session (Guo et al., 2021a). To control the 20-min balance training protocol, the PL group was required to perform the same drills as the BP group. However, unlike the BP group that undertook all the exercises under unstable conditions (i.e., BOSU ball, Swiss ball, and Balance pad), the PL group practiced on the floor. Before the commencement of the study and the initiation of testing, all players completed a 2-week trial period (three sessions/week) to become acquainted with the physical training programs during the formal experimental course of the study. A detailed description of the balance and plyometric training protocols and the biweekly progression are presented in Tables 1, 2. During each session, players received consistent instructions from certified strength and conditioning coaches on proper techniques for agility drills, balance exercises, plyometric exercises, and landing. All the protocols were designed and supervised by one of the authors, who is an experienced researcher in strength and conditioning, and a fitness trainer with a master degree in strength and conditioning.

**TABLE 1:** The balance training program for the BP (combined training) group.

Exercises	The first stage (1–4 weeks)	The second stage (5–8 weeks)	The third stage (9–12 weeks)
Stand on the balance board exercise	Static standing on the board with two legs (3 sets: 30s/set)	Static standing on the board with two legs and eyes closed (3 sets: 30s/set)	Squat on the plate with eyes closed (3 sets: 10 reps/set)
Supine straight leg bridge on Swiss Ball	Isometric supine straight leg bridge on Swiss Ball (3 sets: 30s/set)	Isometric supine single-leg bending bridge on Swiss Ball (3 sets: 30s/set)	Dynamic supine single-leg bending bridge on Swiss (3 sets: 10 reps/set)
Side-plank with inflated balance disc	Side-plank with inflated balance disc with elbow (3 sets: 30s/set)	Side-plank with inflated balance disc and the non-supporting leg stretches backwards (3 sets: 10 reps/set)	Side-plank with inflated balance disc and the non-supporting leg stretches backwards with elastic band (3 sets: 10 reps/set)
Lunge squat on BOSU ball	Lunge squat on BOSU ball (3 sets: 10reps/leg/set)	Lunge squat on BOSU ball and inflated balance disc (3 sets: 10reps/leg/set)	Lunge squat on BOSU ball and inflated balance disc with 5 kg dumbbells (3 sets: 10 reps/leg/set)
Airex^®^ Balance-pad Elite exercise	Single-leg squat with balance-pad (3 sets: 10reps/leg/set)	Single-leg standing with balance-pad and the non-supporting leg stretches backwards (3sets: 12reps/leg/sets)	Single-leg support with balance-pad elite and the non-supporting leg stretches backwards with elastic band (3sets: 12reps/leg/sets)
Rest	Between exercise: 60 s Between sets: 3 min		

**TABLE 2:** The plyometric training program for BP (combined training) and PL (plyometric training) group.

Exercises	The first stage (1–4 weeks)	The second stage (5–8 weeks)	The third stage (9–12 weeks)
Front barrier jump (6 hurdles)	Double-leg front barrier jump (15 cm) (3 sets: 10 reps/set)	Single-leg front barrier jump (15 cm) (3 sets: 5 reps/leg/set)	Single-leg front barrier jump (30 cm) (4 sets: 5 reps/leg/set)
Lateral high-knees with hurdles	4-hurdle (15 cm) (3 sets: 2 reps/set)	6-hurdle (30 cm) (3 sets: 4 reps/set)	6-hurdle (30 cm) (3 sets: 6 reps/set)
Lateral barrier jump	Double-leg jump (15 cm) (3 sets: 10 reps/set)	Double-leg jump (30 cm) (3 sets: 12 reps/set)	Single-leg jump (30 cm) (3 sets: 15 reps/leg/set)
Depth jump	Jump with 20 cm box (3 sets: 8 reps/set)	Jump with 30 cm box (3 sets: 8 reps/set)	Jump with 40 cm box (3 sets: 8 reps/set)
Multi-direction jumps with hurdles	Triangle jump with double-leg (3 hurdles) (3 sets: 6*3 reps/set)	Square jump with single-leg (4 hurdles) (3 sets: 8*3 reps/set)	Hexagon jump with single-leg (6 hurdles) (3 sets: 12*3reps/set)
Intensity and number of contact with ground	Low intensity 144	Middle intensity 234	High intensity 325
Rest	Between exercise: 60 s Between sets: 3 min		

#### Assessment of dynamic balance and quickness

The dynamic balance and lower extremity injury risk were assessed at baseline and within 3 days after the last session of the 12-week training. The tests consisted of dynamic posture stability test and single-legged hop test. All tests were completed within a day. Before each testing session, participants finished warming-up, including a 5-min dynamic stretch, 8-min movement integration, and 2-min neural activation. A 5–10-min rest was given between each test. Each type of test was conducted at the same time and place across different visits, and the participants were asked to wear the same preferred sporting shoes through all the assessments. The players maintained their normal routine of diet and were prohibited from consuming beverages containing caffeine or alcohol during the whole period. The detailed assessments were as described below.

#### Dynamic posture stability test

The test was used to assess the dynamic balance of players through the dynamic posture stability index and center of pressure (Sell, 2012) and is a reliable and sensitive measure of dynamic postural control test (Kiers et al., 2022). Participants stood on an in-ground force plate (Kistler 9281CA, KISTLER, Winterthur, Switzerland, 1,000 Hz) and then jumped anteriorly or laterally with a dominant leg standing for 10 s. The distance between the jumping line and the center of the force plate was 40% of the players’ height (cm) (Sell, 2012; Bourgeois et al., 2017). The fence was placed at the midpoint of the connection between these. The fence height in the forward jump and lateral jump was 30 cm and 15 cm, respectively. All players were asked to complete two types of jumps three times, and the average was considered for data analysis. Matlab software (r2014b, MathWorks, Natick, Massachusetts, USA) was used to calculate the dynamic postural stability index (*DPSI*) and center of pressure (COP). Time-series data for ground reaction force (*GRF*) and center of pressure (COP) were collected within 10 s after the players landed on the force plate with a single leg. All data were smoothed through low-pass filtering, and the truncation frequency was set to 13.33 Hz.

*DPSI* was calculated from the *GRF* curve within 3 s after touchdown (the time when the *GRF* value exceeded 5% of body weight) (Wikstrom et al., 2005). Where *BW* is the body weight, *GRF*_*x*_, *GRF*_*y*_, and *GRF*_z_ are the back and forth, left and right, and vertical ground reaction forces. The dynamic posture stability indexes of forward jump (DF-DPSI) and lateral jump (DL-DPSI).

$$DPSI=\frac{\left(\frac{\sqrt{\sum {\left(0-GRF_{x}\right)}^{2}+\sum {\left(0-GRF_{y}\right)}^{2}+\sum {\left(BW-GRF_{z}\right)}^{2}}}{\;\text{number}\;\text{of}\;\text{data}\;\text{points}\;}\right)}{BW}$$

COP was calculated from the time series within 10 s after landing, and the back and forth displacement difference (COP_AP_), left-to-right displacement difference (COP_ML_), and total displacement distance (COP_PL_) of the forward jump (DF) and lateral jump (DL) were calculated (Ziv and Lidor, 2010b). X_T_ and Y_T_ are the back and forth, and left and right displacements at T seconds, and the value of T is 1–10 s.

$${\text{COP}}_{\text{AP}}=\sum_{1}^{10}\;{\left(x_{t}-\bar{x}\right)}^{2}$$

$${\text{COP}}_{\text{PL}}=\sum_{0}^{9}\;\sqrt{{\left(x_{t+1}-x_{t}\right)}^{2}+{\left(y_{t+1}-y_{t}\right)}^{2}}$$

#### Lower extremity injury risk test

**FIGURE 2:**
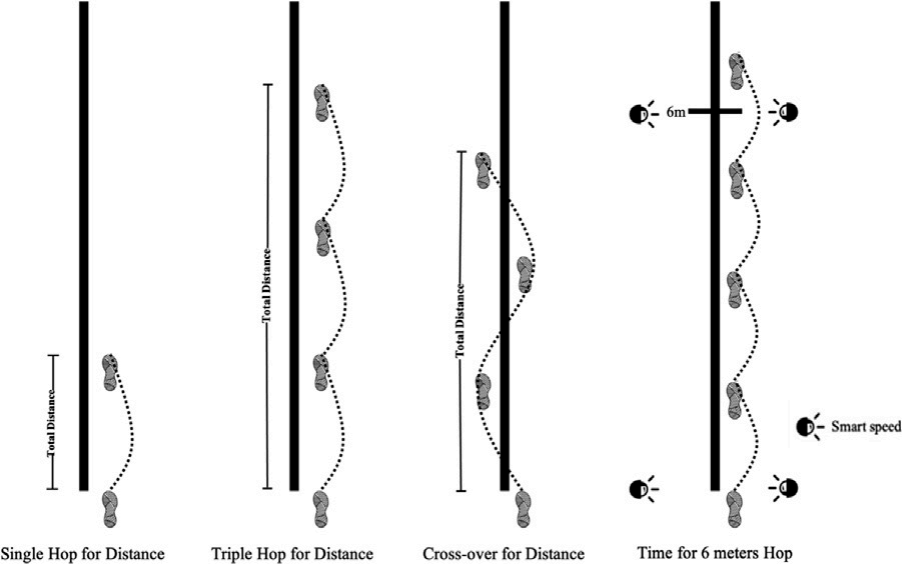
Single-legged hop tests.

Single-legged hop tests can serve as a predictive factor of knee function in individuals to evaluate the risk of ACL injury and discriminate between individuals who return to their previous activity level after ACL injury or reconstruction (Figure 2). The single hop for distance, triple hop for distance, cross-over hop for distance, and time for the 6 m hop were measured. Smart Speed device (Fusion Sport, Coopers Plains, Australia) was set to record the time for the 6 m hop. After hopping, participants needed to stand with a single leg for 2 s to make the results effective. Participants were asked to jump three times on every leg in each test, and the longest distance and the shortest time in the three tests were taken as the final data when the four tests’ lower LSI was calculated. LSI was counted as the ratio between the non-dominant leg (N) and the dominant leg (D), while the 6 m hop time was calculated from the dominant and non-dominant leg in the division. Four types of LSI were defined, including LSIO (Single Hop for Distance), LSIT (Triple Hop for distance), LSIC (Cross-over for distance), and LSIS (Time for 6 m Hop) in this study. When LSI ≥85%, there is no risk of ACL injury; and when LSI <85%, ACL is at risk of injury (Noyes et al., 1991).

### Statistical analysis

Experimental data were processed using IBM SPSS statistical software package (version 25.0, IBM, Chicago, IL, United States). All data were presented as means and SD. The level of significance was set at p < 0.05 for all tests. To examine the effects of the combined training on the performance of single-legged hop tests and proprioception tests, we first performed two-way repeated-measure ANOVA (group × time). The dependent variable for each model was LSIO, LSIT, LSIC, LSIS, D-(AP, ML, LSI), and N-(AP, ML, LSI). The model factors were group, time, and their interaction. When a significant interaction was observed, LSD post-hoc correction was performed to identify the location of the significance. Secondly, we examined the effects of CT on the performance within each group, as well as the percentage changes from pre-to post-intervention between CT and PT by using separate one-way ANOVA models. The model factor was time. Partial η^2^ was used to assess the effect size (ES) where significance was observed, with its strength being interpreted as the following: <0.06 as small, <0.14 as moderate, and ≥0.14 as large.

## Results

All participants completed this study and were included in the analysis. All the data were normally distributed (*p* > 0.207). There were no significant differences in demographic characteristics (i.e., age, body weight, and height), primary outcomes (i.e., LSI and DPSI), and secondary outcomes (i.e., COP) between the PB and PL groups (*p* > 0.947).

**FIGURE 3:**
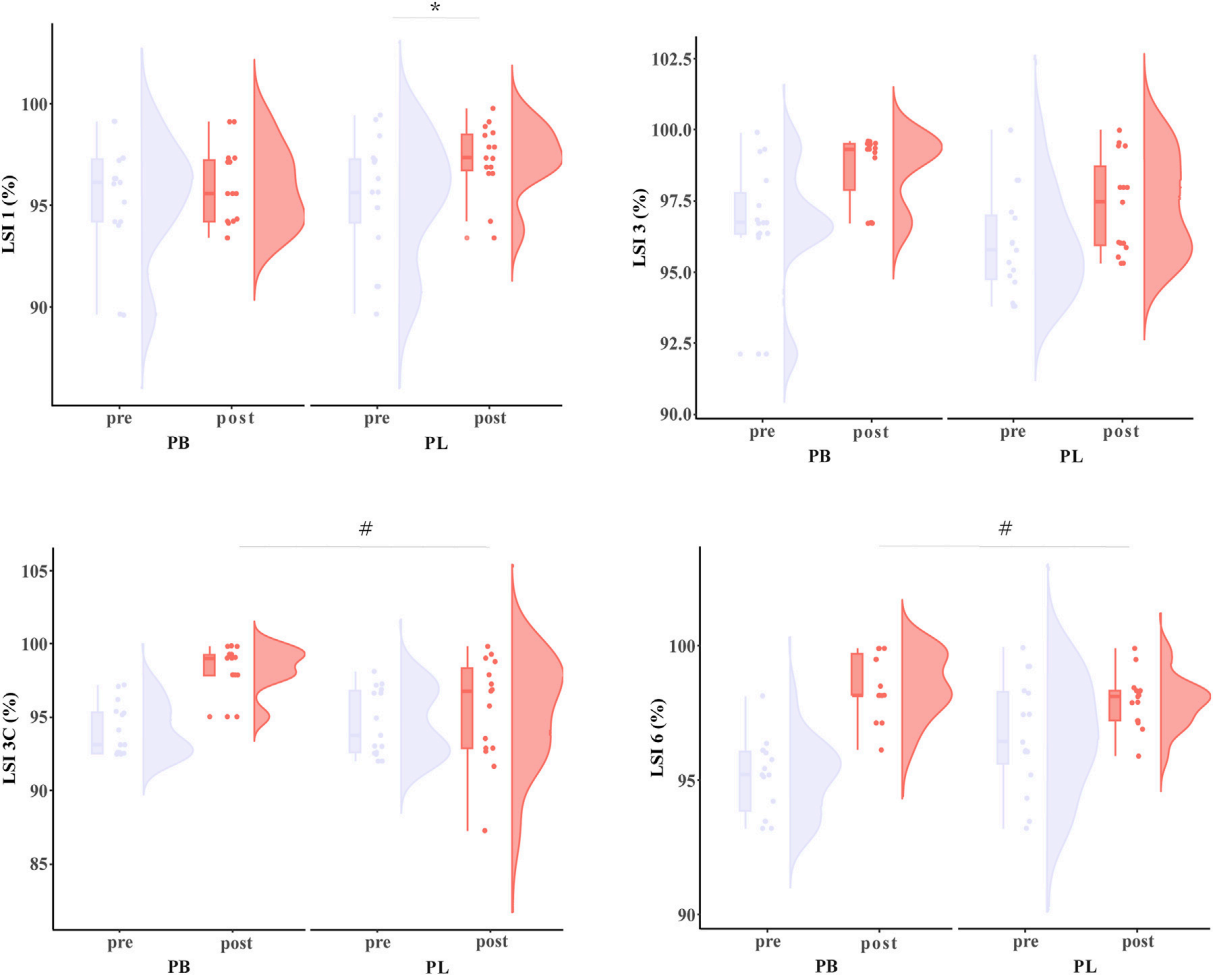
The LSI results for BP (combined training) and PL (plyometric training) before and after the 8-week training. * Statistically significant difference between pre-and post-test, *p* < 0.05; # Statistically significant difference between group, *p* < 0.05.

Primary two-way repeated-measures ANOVA models showed significant time effect and interactions between group and time on LSI3C (*p* < 0.001) and LSI6 (*p* = 0.006). Post hoc analysis revealed that LSI3C (*p* < 0.003) and LSI6 (*p* < 0.027) were significantly greater after the PB intervention compared to all the other pre- and post-interventions. Additionally, primary two-way repeated-measures ANOVA models also showed significant time effect on LSI3 (*p* < 0.001). The exploratory ANOVA model showed that within both the PB and PL groups, LSI3 (PB: *p* = 0.004; PL: *p* = 0.043) was significantly improved after the intervention as compared to baseline (Table 3; Figure 3).

**TABLE 3:** The assessment results for BP (combined training) and PL (plyometric training) before and after the 8-week training.

	PB group		PL group		Time effect		Time × Group interaction effect	
	Pre	Post	Pre	Post	*P* value	Partial $\eta^{2}$	*P* value	Partial $\eta^{2}$
LSI 1 (%)	95.44 ± 2.83	95.83 ± 1.92	95.41 ± 3.01	97.30 ± 1.72^a^	0.076	0.055	0.236	0.025
LSI 3 (%)	96.70 ± 1.23	98.67 ± 1.71	95.98 ± 1.82	97.32 ± 2.22	<0.001	0.189	0.493	0.008
LSI 3C (%)	94.13 ± 1.77	98.32 ± 1.54#	94.61 ± 2.26	95.49 ± 3.56	<0.001	0.229	0.010	0.112
LSI 6 (%)	95.13 ± 1.40	98.54 ± 1.19#	96.70 ± 2.09	97.93 ± 1.00	<0.001	0.398	0.006	0.128
DF-DPSI	0.387 ± 0.001	0.378 ± 0.005#	0.387 ± 0.002	0.383 ± 0.002	<0.001	0.625	<0.001	0.224
DL-DPSI	0.385 ± 0.002	0.376 ± 0.005#	0.385 ± 0.001	0.382 ± 0.002	<0.001	0.559	<0.001	0.264
NF-DPSI	0.389 ± 0.002	0.374 ± 0.007#	0.388 ± 0.002	0.384 ± 0.002	<0.001	0.626	<0.001	0.312
NL-DPSI	0.386 ± 0.002	0.374 ± 0.007#	0.386 ± 0.001	0.380 ± 0.005	<0.001	0.552	0.009	0.114

^a^ Statistically significant difference between pre-and post-test, p < 0.05; # Statistically significant difference between group, p < 0.05.

**FIGURE 4:**
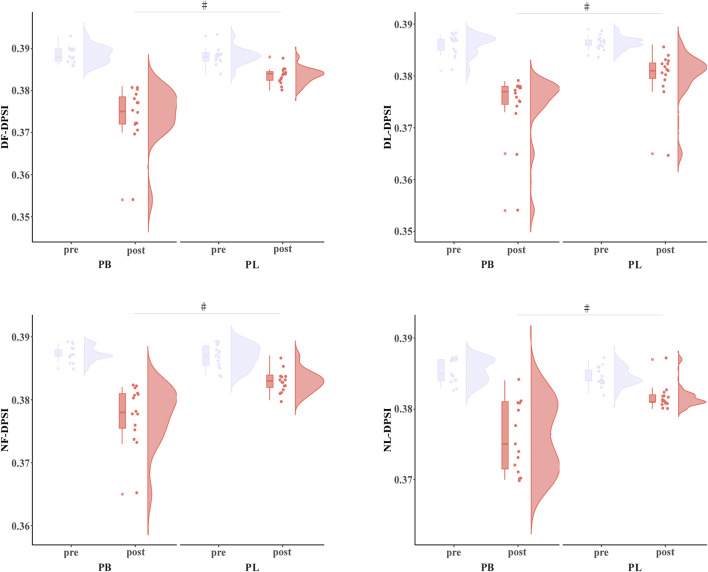
The DPDI results for BP (combined training) and PL (plyometric training) before and after the 8-week training. * Statistically significant difference between pre-and post-test, *p* < 0.05; # Statistically significant difference between group, *p* < 0.05.

The primary two-way repeated-measures ANOVA models showed significant time effect and interactions between group and time on DF-DPSI (*p* < 0.001), DL-DPSI (*p* < 0.001), NF-DPSI (*p* < 0.001), and NL-DPSI (*p* = 0.009). Post hoc analysis revealed that DF-DPSI (*p* < 0.001), DL-DPSI (*p* < 0.007), NF-DPSI (*p* < 0.002), and NL-DPSI (*p* < 0.001) were significantly greater after the PB intervention compared to all the other pre- and post-interventions (Table 3; Figure 4).

**FIGURE 5:**
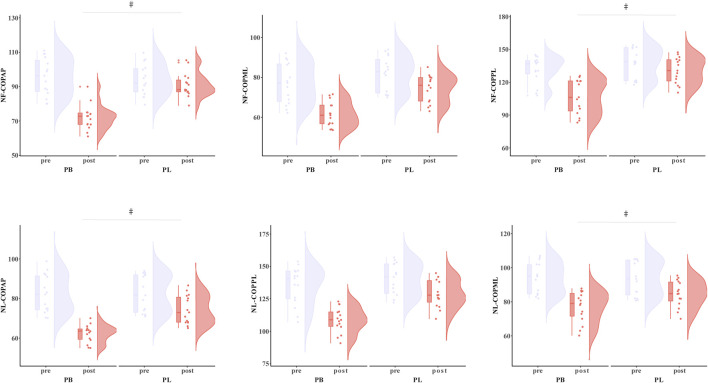
The NF-COP results for BP (combined training) and PL (plyometric training) before and after the 8-week training. * Statistically significant difference between pre-and post-test, *p* < 0.05; # Statistically significant difference between group, *p* < 0.05.

The primary two-way repeated-measures ANOVA models showed significant time effect and interactions between group and time on DF-COP_AP_ (*p* = 0.015), DL-COP_AP_ (*p* = 0.019), NF-COP_AP_ (*p* < 0.001), NL-COP_AP_ (*p* = 0.006), DF-COP_PL_ (*p* = 0.029), DL-COP_PL_ (*p* = 0.028), NF-COP_PL_ (*p* = 0.013), and NL-COP_PL_ (*p* = 0.015). Post hoc analysis revealed that DF-COP_AP_ (*p* < 0.038), DL-COP_AP_ (*p* < 0.017), NF-COP_AP_ (*p* < 0.001), NL-COP_AP_ (*p* = 0.007), DF-COP_PL_ (*p* < 0.001), DL-COP_PL_ (*p* < 0.007), NF-COP_PL_ (*p* < 0.001), and NL-COP_PL_ (*p* < 0.015) were significantly greater after the PB intervention compared to all the other pre- and post-interventions (Table 4; Figure 5).

**TABLE 4:** The assessment results for BP (combined training) and PL (plyometric training) before and after the 8-week training.

	PB group		PL group		Time effect		Time × Group Interaction effect	
	Pre	Post	Pre	Post	*P* value	Partial $\eta^{2}$	*P* value	Partial $\eta^{2}$
DF-COP_AP_	90.29 ± 12.2	72.19 ± 7.39#	95.06 ± 8.63	88.27 ± 5.24	<0.001	0.352	0.015	0.101
DF-COP_ML_	72.76 ± 11.35	60.55 ± 7.09	81.94 ± 8.75	74.81 ± 8.13	<0.001	0.238	0.277	0.021
DF-COP_PL_	131.56 ± 15.27	109.66 ± 16.64#	137.27 ± 13.8	132.19 ± 12.05	<0.001	0.187	0.029	0.082
DL-COP_AP_	79.29 ± 9.97	61.23 ± 4.40#	82.45 ± 9.41	74.88 ± 8.79^a^	<0.001	0.382	0.019	0.094
DL-COP_ML_	90.71 ± 9.68	81.37 ± 9.63	93.66 ± 10.07	85.17 ± 7.77	<0.001	0.196	0.859	0.001
DL-COP_PL_	131.25 ± 15.18	104.72 ± 9.28#	139.82 ± 12.39	127.44 ± 11.09	<0.001	0.406	0.028	0.083
NF-COP_AP_	95.58 ± 10.92	72.09 ± 7.23#	93.80 ± 9.04	90.89 ± 7.03	<0.001	0.382	<0.001	0.273
NF-COP_ML_	76.93 ± 10.18	61.40 ± 6.25	81.94 ± 8.75	74.52 ± 6.88	<0.001	0.346	0.060	0.062
NF-COP_PL_	131.54 ± 12.43	107.09 ± 15.76#	137.27 ± 13.80	130.79 ± 11.90	<0.001	0.258	0.013	0.105
NL-COP_AP_	82.26 ± 9.62	62.25 ± 4.44#	82.45 ± 9.41	74.30 ± 7.24	<0.001	0.456	0.006	0.130
NL-COP_ML_	93.74 ± 9.07	77.12 ± 9.07	93.66 ± 10.07	84.74 ± 7.68	<0.001	0.350	0.103	0.047
NL-COP_PL_	135.19 ± 14.69	108.64 ± 9.64#	139.82 ± 12.39	128.87 ± 10.59	<0.001	0.396	<0.001	0.102

^a^ Statistically significant difference between pre-and post-test, *p* < 0.05; # Statistically significant difference between group, *p* < 0.05.

**FIGURE 6:**
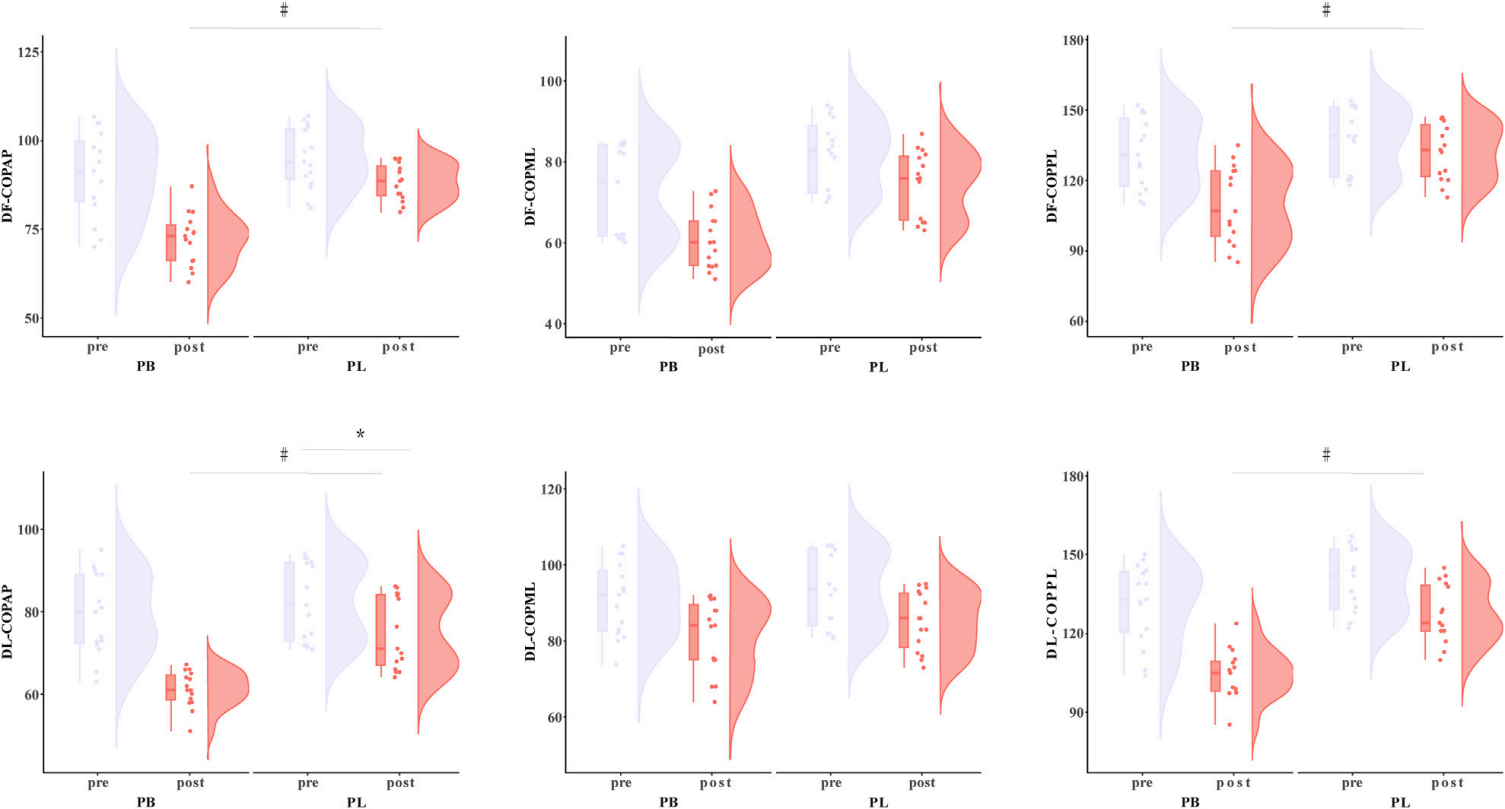
The DF-COP results for BP (combined training) and PL (plyometric training) before and after the 8-week training. * Statistically significant difference between pre-and post-test, *p* < 0.05; # Statistically significant difference between group, *p* < 0.05.

The primary two-way repeated-measures ANOVA models showed significant time effect on DF-COP_ML_, DL-COP_ML_, NF-COP_ML_, and NL-COP_ML_. The exploratory ANOVA model showed that within both the PB and PL groups, DF-COP_ML_ (PB: *p* < 0.001; PL: *p* = 0.034), DL-COP_ML_ (PB: *p* = 0.008; PL: *p* = 0.016), NF-COP_ML_ (PB: *p* < 0.001; PL: *p* = 0.016), and NL-COP_ML_ (PB: *p* < 0.001; PL: *p* = 0.009) were significantly improved after the intervention as compared to baseline (Table 4; Figure 6).

## Discussion

The aim of the present study was to investigate the effect of combined balance and plyometric training on dynamic balance ability and injury risk in college dancers. To our knowledge, this is the first study to explore the effect of BP in dancers. The results of the present study showed that BP significantly enhanced the LSI-3C, LSI-6, and indicts of DPSI, indicating that BP is more effective in improving the dynamic balance ability and reducing lower limb injury risk.

At a competitive level, dance performance is not a single art activity, but it is rather a complex phenomenon that relies on various elements with both direct and indirect effects, such as physically fitness. Dancing involves a lot of quick and slow motions, including acceleration, deceleration, rotation, and single leg support (Clarke et al., 2021), and this makes dynamic balance a key component of fitness training (Koutedakis and Jamurtas, 2004). Previous studies have demonstrated the effectiveness of BP in improving dynamic balance among athletes in different sports, such as basketball (Guo et al., 2021b), badminton (Lu et al., 2022), and taekwondo (Shen, 2024). Our study further confirms the effectiveness of BP in improving the balance ability of dancers.

The results of this study indicated that BP induced a significant improvement in the DPST performance compared with PL. BP significantly improved all DPSI indices, whereas PL did not. In a previous study, participants with functional ankle instability exhibited poor ankle joint reposition sense (Cho and Park, 2019). Both balance training on an unstable surface and plyometric training could activate the mechanical or proprioception receptor on foot or tendon ligaments around the ankle joint (Docherty et al., 1998; Lin et al., 2021). This may explain the improved dynamic balance observed in our study due to enhanced ankle joint reposition sense. The lack of significant improvement in DPSI with PL training alone may be attributed to the dancers’ relatively weak strength, suggesting that additional strength training is necessary to achieve the benefits of PL training.

Additionally, our findings reveal that BP significantly reduced COP displacement in the anterior-posterior direction during single-leg landing (DF-COP_AP_, DL-COP_AP_, NF-COP_AP_, and NL-COP_AP_ all showed significant improvements compared to PL). This finding is inconsistent with a previous study which showed that the integration of plyometric and core stability training reduced the COP displacement in the medial-lateral direction (Lin et al., 2021). This discrepancy may be due to differences in testing methods. The present study used a dynamic postural stability test, whereas the other study employed specific ballet movements. The direction and distance of the jumps in these studies could have contributed to the varying results. The balance training program in our study included numerous core stability training, particularly performed on unstable surfaces (such as BOSU balls and Swiss ball). A previous study demonstrated that improved core stability could reduce the range of COP displacements (Kaji et al., 2010). This likely resulted in enhanced co-contraction of the hip and core muscles, thereby improving the dynamic balance capabilities. Additionally, the plyometric training could effectively stimulate the neural reflexes of the ankle joint, thereby enhancing postural adjustments in the anterior-posterior direction (Winter et al., 1996). The improved postural control may also be due to an enhanced reposition sense of the ankle joint and core stability. Compared with PL, BP significantly improved LSI 3C and LSI 6, while PL significantly improved LSI 1. LSI-3C reflects the capacity to generate forces in the frontal and transverse planes with multiple hops in the sagittal plane (Logerstedt et al., 2012). It requires a high level of stretch-shortening cycle (SSC) function and good postural control, both of which are crucial skills in dance performance. Traditional plyometric training focuses solely on enhancing SSC function and does not address improvements in balance, particularly trunk dynamic balance, during rapid lateral movements. The BP training could integrate both balance training and plyometric training, and our result demonstrated that this combination could reduce the limb asymmetry during continuous lateral movement. Besides, LSI 6 was also improved in our study. LSI 6 can be used to identify the dynamic stability of knees in ACL injury rehabilitation (Zwolski et al., 2016). Unlike LSI 3, LSI 6 represents a greater number of consecutive hops, where balance may play a more significant role in the later hops. Dance often involves continuous, rapid forward and backward movements (Bird, 2016), which closely resemble the movement pattern assessed by LSI-6. A previous study demonstrated that there was greater instability on the non-dominant side and more stability on the dominant side (Kilroy et al., 2016), leading to high injury risk during high technical demand movement. The present study showed that BP could significantly reduce the asymmetry between the dominant and non-dominant limb, thereby reducing the injury risk in dance performance.

The findings of this study suggest that incorporating BP training into dance training programs can significantly improve dynamic balance and reduce the risk of lower limb injuries. Dance instructors and physical trainers should consider integrating balance and plyometric exercises to enhance the performance and safety of dancers. Future research should aim for larger sample sizes and longer training durations to validate these findings. Additionally, exploring the effects of BP training on other aspects of dance performance, such as agility and muscle strength, can provide a more comprehensive understanding of its benefits. We believe that the incorporation of this training protocol into a dancer’s regular training routine over the course of their career could significantly reduce injury risk, which should be investigated in future longitudinal studies. Furthermore, given that the balance demands for dancers are generally higher than those required in many other sports, such as team sports like soccer and basketball, or racket sports like badminton and tennis, the findings of this study may also have potential applications in these areas. However, the specific effects in these contexts still require further investigation. Several limitations of this study should be noted. First, the assessment metrics used in this study primarily reflect general balance and injury risk indicators, rather than tests directly related to specific dance activities. Second, the injury risk in this study was predicted using LSI, which does not directly reflect the actual risk of lower limb injuries. Longer-term observations are needed to obtain real injury data and validate the predictive power of LSI in this context.

## Conclusion

The 12-week combined balance and plyometric training program was more effective than plyometric training alone in improving dynamic balance and reducing lower extremity injury risk in college dancers. This combined training approach is recommended for improving performance and preventing injuries in dancers.

## Data Availability

The original contributions presented in the study are included in the article/supplementary material, further inquiries can be directed to the corresponding author.
