# Corrigendum: The effect of 12-week combined balance and plyometric training on dynamic balance and lower extremity injury risk in college dancers

**DOI:** 10.3389/fphys.2025.1575609

**Published:** 2025-04-03

**Authors:** Yuqi Yan, Park Seoyoung, Heo Seomyeong, Yi Zhao

**Affiliations:** ^1^ Dance College, Sichuan Normal University, Chengdu, China; ^2^ School of College of Art and Physical Education, Hanyang University, Seoul, Republic of Korea

**Keywords:** dynamic balance, plyometric training, balance training, lower extremity injury risk, college dancers dynamic balance, college dancers

In the published article, there was an error in affiliation 1. Instead of “School of Dance, Sichuan Normal University, Chengdu, China”, it should be “Dance College, Sichuan Normal University, Chengdu, China.”

In the published article, there was an error. The **Conclusion** included incorrect text.

A correction has been made to **Conclusion**, paragraph 1. This sentence previously stated:

“This pilot study showed that CT is of great promise to induce significantly greater improvements in strength and power of firefighters compared to RT, thereby better enhancing their capabilities for occupational activity. The knowledge obtained from this study will ultimately help inform the design of future larger-scale studies to confirm the findings in this study and help firefighter agencies to develop more appropriate fitness training and management programs for firefighters in their daily routine.”

The corrected sentence appears below:

“The 12-week combined balance and plyometric training program was more effective than plyometric training alone in improving dynamic balance and reducing lower extremity injury risk in college dancers. This combined training approach is recommended for improving performance and preventing injuries in dancers.”

The authors apologize for this error and state that this does not change the scientific conclusions of the article in any way. The original article has been updated.

